# Microscopic Techniques for the Analysis of Micro and Nanostructures of Biopolymers and Their Derivatives

**DOI:** 10.3390/polym12030512

**Published:** 2020-02-27

**Authors:** Abhilash Venkateshaiah, Vinod V.T. Padil, Malladi Nagalakshmaiah, Stanisław Waclawek, Miroslav Černík, Rajender S. Varma

**Affiliations:** 1 Department of Nanomaterials in Natural Sciences, Institute for Nanomaterials, Advanced Technology and Innovation, Technical University of Liberec, 461 17 Liberec, Czech Republic; abhilash.venkateshaiah@tul.cz (A.V.); stanislaw.waclawek@tul.cz (S.W.); 2 IMT Lille Douai, Department of Polymers and Composites Technology and Mechanical Engineering (TPCIM), 941 rue Charles Bourseul, CS10838, F-59508 Douai, France; 3 Regional Centre of Advanced Technologies and Materials, Department of Physical Chemistry, Faculty of Science, Palacký University in Olomouc, Šlechtitelů 27, 783 71 Olomouc, Czech Republic

**Keywords:** biopolymers, microstructures, nanostructures, surface morphology, filler dispersion, chemical composition, optical microscopy, scanning electron microscopy, transmission electron microscopy, atomic force microscopy

## Abstract

Natural biopolymers, a class of materials extracted from renewable sources, is garnering interest due to growing concerns over environmental safety; biopolymers have the advantage of biocompatibility and biodegradability, an imperative requirement. The synthesis of nanoparticles and nanofibers from biopolymers provides a green platform relative to the conventional methods that use hazardous chemicals. However, it is challenging to characterize these nanoparticles and fibers due to the variation in size, shape, and morphology. In order to evaluate these properties, microscopic techniques such as optical microscopy, atomic force microscopy (AFM), and transmission electron microscopy (TEM) are essential. With the advent of new biopolymer systems, it is necessary to obtain insights into the fundamental structures of these systems to determine their structural, physical, and morphological properties, which play a vital role in defining their performance and applications. Microscopic techniques perform a decisive role in revealing intricate details, which assists in the appraisal of microstructure, surface morphology, chemical composition, and interfacial properties. This review highlights the significance of various microscopic techniques incorporating the literature details that help characterize biopolymers and their derivatives.

## 1. Introduction

The ever-rising concern about the environmental impact of synthetic polymers has stimulated a great deal of research interest in the polymers of biological origins. The combination of sustainability and biodegradability of biopolymers is the reason they are gaining precedence over the fossil fuel-derived polymers [1]. Historically, biopolymers had commanded a lot of importance in various applications such as food, clothing, ropes, and furniture [2]. However, since the discovery of synthetic polymers, these biopolymers were replaced. With the increasing public awareness of global warming and the environmental toll as well as the rapidly depleting fuel sources, these biomaterials are emerging as a potential replacement for conventional polymers; during the last two decades, this interest has been the driving force in the development of sustainable biopolymers from renewable sources [3,4,5].

Biopolymers encompass a wide range of materials derived from biological sources like plants, animals, trees, microorganisms, and also materials synthesized from other sources including sugars, proteins, vegetable oils, fats, resins, and exudate [6]. The common distinguishable property with all these biomaterials is that, given a definite time, they break down to form simple molecules such as carbon dioxide and water under the enzymatic action of microorganisms. In comparison with conventional polymers with a simpler structure, nature-derived biopolymers have a wide variety of structural complexities, often reliant on many factors including the source, their species, the age of the plant, and the method of extraction.

Biopolymers, owing to their versatile nature have tremendous potential to replace conventional polymers in a wide range of applications including packaging, textiles, cosmetics, food technology, drug delivery, and structural materials [6]. However, the commercialization of biopolymers is hampered by shortcomings of economic and engineering aspects. Competition with their cheaper synthetic counterparts, lower mechanical strength, and high hydrophilicity impede their industrial use. Several techniques have been developed to overcome these drawbacks including chemical modification [7,8], blending with other biodegradable polymers [9,10], and by using additive fillers [11,12] and plasticizers [13]. Recently, biopolymer research crossed paths with nanotechnology and the resulting materials have been making tremendous progress. The advent of nanotechnology in the biopolymer avenue has opened many potential doors. In combination with the nanomaterials, biopolymers display superior properties when compared with unblended ones. The intersection of the fields of biopolymers and nanotechnology has been in more than one form; biopolymers blending with nanomaterials offered nanocomposites [14,15], besides being made into nanoparticles [16,17] and nanofibers [18,19,20]. Additionally, biopolymers have also been used as reducing and stabilizing agents in the generation of nanoparticles [21,22,23,24,25]. The fusion of these two fields has paved the path for a whole class of materials, which not only have the potential to replace conventional fossil fuel-based polymers but also are environmentally benign.

Contemporary research ensued regarding the emergence of various classes of materials; hence, proper characterization is necessary to gain insights into their complex structure and morphology to determine their ideal applications. Microscopy techniques represent a class of multifunctional techniques often used for the in-depth analysis and understanding of materials over a large-scale magnification range. Microscopy is the observation of materials and their properties in the range of millimeters to nanometers, which are not visible to the naked eye. Observation of the materials at such high magnifications can provide physical, chemical, and structural information, which are associated with the performance of the material. Microscopy techniques can provide the necessary information by acquiring an image of the material and the morphology of the sample is analyzed with the help of this image at micro or nanoscale. Different microscopic techniques have been developed over the years ranging from optical microscopes to electron microscopes and scanning probe microscopes for the analysis of material morphology on varying length scales.

Microscopy techniques have been a prominent part of the material research over decades and have aided in numerous discoveries. The unprecedented importance of their contributions in the field of material research can be recognized by the fact that most research articles provide a direct or indirect reference to their use. With the increased focus on biopolymers in the recent past, the use of microscopic techniques for their analysis has been an invaluable asset in aiding researchers’ innate need to understand these natural wonder materials. The aim of this article is to accentuate various microscopic techniques often used to investigate the bio-polymeric systems and their influence on current research. This article provides a compilation of the assortment of microscopic techniques used in the field of biopolymers along with an introduction to their mechanism of operation. Furthermore, the article emphasizes the significance of microscopic techniques in the elucidation of various micro and nanostructures of biopolymers. Additionally, several leading articles have been cited in the present review, which will aid the researchers to comprehend the recent development of various microscopic techniques deployed.

## 2. Biopolymers

**Figure 1:**
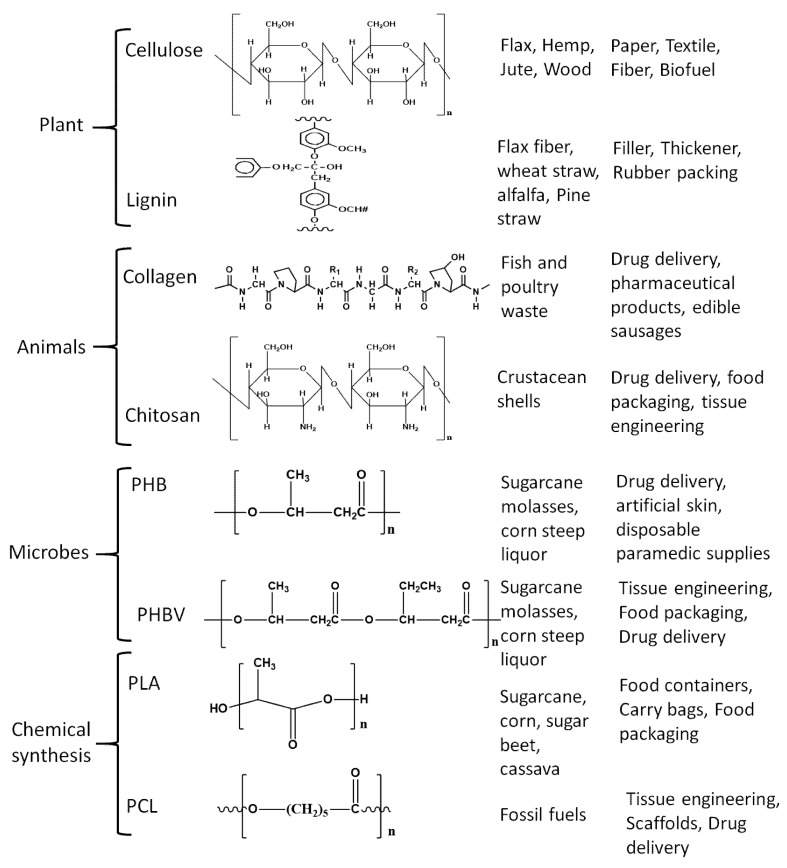
Common biopolymers with their structure, source, and applications.

Polymers generated from renewable resources or polymers that degrade into carbon dioxide and water under certain conditions in a limited time are termed biopolymers and are classified into distinct types as shown in Figure 1 [26,27,28]. Since different criteria are followed in the literature for their classification, herein, we have briefly discussed assorted biopolymers, their classification, and applications.

Biopolymers are mainly divided into two types as natural or synthetic/manmade polymers; further, they are divided into subsections depending on their manufacturing process and inherent functional groups. Natural biopolymers are further sub-classified into two types based on the sources, as plant-derived or animal-derived biopolymers; plant-based biopolymers are those which are obtained from plants, trees, or biomass and are called agro-polymers, such as cellulose, starch, hemicellulose, and lignin, among others [27,28]. Cellulose is the most abundantly available polymer in today’s world with around 1.5 × 10^12^ tons of annual production and is considered an inexhaustible source of biopolymer [29], often extracted from various agricultural and other natural sources [30,31]. Cellulose contains long chains of unbranched β (1→4) linked D- glucopyranosyl units and the chain length of β (1→4) glucan depends on factors including the species of the plant, growth environment, and maturity. Cellulose has found major applications in paper, textiles, and fiber industries, and even as a biofuel source. Lignin is the second most naturally available biopolymer after cellulose with a highly branched structure bearing various oxygenated functional groups like hydroxyl, carboxyl, carbonyl, and methoxyl groups [32,33]. Lignin comprises 15%–40% of dry matter in woody plants and consists of three phenyl propane units also called monolignols namely, coniferyl-, sinapyl-, and *p*-coumaryl alcohol [34]. Lignin has been used as a rubber intensifier, filler in composite materials, in rubber packing, thickener for paints and coatings [34]. Starch is another abundant polysaccharide extractable from agricultural raw materials, main crops being potatoes, corn, and rice, where starch is produced as granules and stored in seeds and swollen stems. The composition of starch varies from plant to plant but mostly consists of amylose and amylopectin. Amylose is a linear molecule bearing (1→4)-linked α-D-glucopyranosyl units with a very few branched links, while amylopectin has a highly branched structure with (1→4)-linkages of α-D- glucopyranosyl residues and (1→6) linkages at the branch points [35]. Applications of starch include disposable food service wares, food packaging, carrying bags, and loose fill products [36].

Biopolymers derived from animals significantly differ from those derived from plants and include proteins (e. g. collagen) and polysaccharides (e. g. chitosan). Collagen is an abundant protein constituent present in the connective tissues of vertebrate and invertebrate animals with various types identified to date, but Type Ⅰ collagen is the most common and explored. It consists of three polypeptide subunits, each composed of amino acids containing glycine, proline, hydroxyproline, and lysine [37] and is used in pharmaceutical products, drug delivery applications, and edible casings [38]. Chitosan is a random copolymer derived from chitin, a polysaccharide found in the crustacean shells [39] which upon alkaline deacetylation yields chitosan and consists of β-1,4 glycosidic linked D-glucosamine and *N*-acetyl-D-glucosamine units [40]. Chitosan, because of its non-toxic nature, has been used as a food additive, in drug delivery, and owing to its film-forming properties has been deployed in tissue engineering and food packaging [41,42,43]. Additional applications for chitosan are in the beverage industry, as a support for enzyme immobilization, and also as a reinforcing filler [44,45].

Man-made or synthetic biopolymers are derived with human intervention and can be further divided into the biopolymers generated from microorganisms and chemically synthesized polymers, e.g., polyhydroxyalkanoates (PHA), are produced from microorganisms at certain biotechnological (pH and temperature) conditions and specific nutrients [46,47]. Renewable PHA is synthesized by bacteria from such renewable resources as carbon source, and owing to their plastic-like properties combined with biodegradability is viewed as a potential replacement for polyethylene and propylene, with applications in food packaging [48,49]. However, this potential is being encumbered by its poor mechanical properties and brittle nature [48,49]. PHB is the most popular member of the PHA family acquired via fermentation of sugars by the bacteria *Alcaligenes eutrophus.* PHB is non-toxic and degrades in vivo into d3-hydroxybutyric acid, which is commonly found in human blood. Not surprisingly, PHB has been used in heart valves, controlled drug release, artificial skin, and disposable paramedical supplies [50,51]. PHB has inherent poor mechanical properties and efforts are being made to improve those to compete with the conventional polymers [52,53]. Blending is one such endeavor, wherein, PHB family blends are found to be compatible and have enhanced co-crystallization as exemplified by poly (3-hydroxybutyrate-co-3-hydroxyvalerate), (PHBV) which is a blend of PHB and hydroxyvaleric acid. This copolymer has less crystallinity, improved flexibility and processability, and is commercially available under the name Biopol.

Chemically synthesized biopolymers include polymers made from monomers (e.g., polylactic acid), from biobased materials, and biopolymers obtained via petro-based monomers (e. g. poly-ε-Caprolactone). Polylactic acid (PLA) is a biodegradable aliphatic thermoplastic polyester prepared from starch as a major source [54,55]. The lactic acid monomer is extracted by enzymatic hydrolysis of starch from corn, tapioca, and sugarcane and is polymerized by polycondensation reaction to get low molecular weight PLA and ring-opening polymerization of lactides to obtain high molecular weight PLA [56]. PLA has zero to low toxicity, is biodegradable, and has mechanical properties comparable to those of conventional commercial polymers [10,57,58]. Among the petro-derived biopolymers, poly-ε-caprolactone (PCL) is the most common and popular biodegradable polyester and is obtained by catalytic ring-opening polymerization of ε-caprolactone [59,60]. Owing to its biodegradability combined with nontoxicity and biocompatibility, it is used in the preparation of scaffolds for tissue engineering and in controlled drug delivery [61,62,63].

## 3. Microscopic Techniques

Currently, a plethora of characterization techniques are available to analyze and characterize materials, and they assist in recognizing the end-use applications of the materials by providing a thorough knowledge of the structure and property relationships. Among these, microscopy techniques command a unique position in analyzing various features such as morphology, chemical composition and structure, topology, interfacial properties, molecular, microstructure, and micromechanical properties.

**Figure 2:**
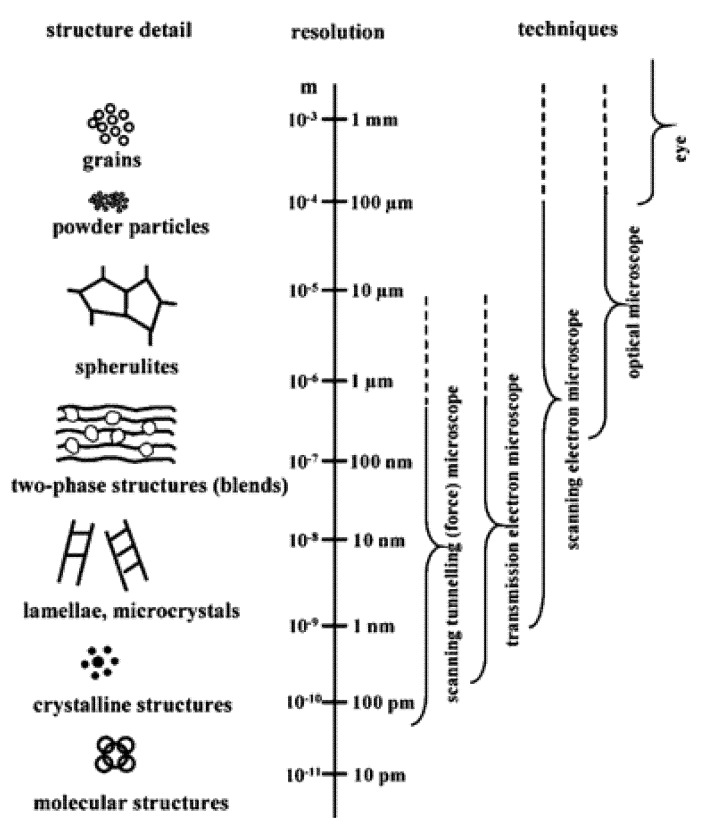
Assorted polymeric structures and the attainable resolutions with various microscopic techniques [64]. (Reprinted with permission from Michler et al., 2008.)

Several microscopy techniques used for the characterization and analysis of diverse biopolymers and their derivatives are discussed in this article with a brief introduction to the mechanism of the microscopic techniques followed by their usage in research (Figure 2 and Table 1).

### 3.1. Optical Microscopy

Optical microscopy is probably the simplest and oldest among the microscopy techniques [88]. It is a two-dimensional imaging technique and since its time of inception, there have been incessant research upgrades to suit modern needs [89,90,91,92]. A simple optical microscope comprises of two converging lenses, an objective, and an eyepiece and utilizes the optical theory of lenses to operate, wherein light emerging from the sample will be collected by the objective and directed towards the eyepiece [93]. Usually, the sample is illuminated by two methods—episcopic (reflected) or diascopic (transmitted). Generally, the reflected techniques provide the essential information, and transmitted light optical microscopy is opted to gain insights into the microstructures, while in some cases etchants, stains, or dyes may be required for entrenched analysis of morphology [94,95,96]. Light detecting devices, namely charge-coupled camera, photodiodes, photomultiplier tubes, and other optical sensors are widely employed to collect the image of the sample. Modern optical microscopic imaging systems are equipped with electron scanning systems like galvano-mirrors, acousto-optic deflectors, or fast confocal illumination systems. It is the simplest of all the other microscopic techniques, as the samples can be directly viewed at a magnification up to 1500× with a theoretical resolution of 200–300 nm in lateral resolution, and 500–700 nm in axial resolution [97].

**Figure 3:**
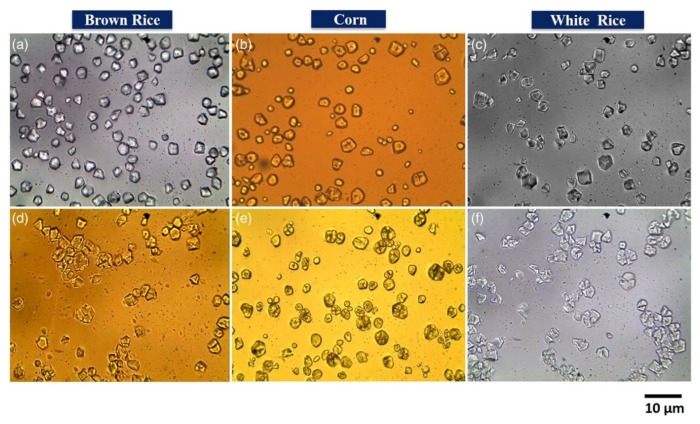
Optical microscopic (40× magnification) images of starch granules of native (**a**) brown rice, (**b**) corn, and (**c**) white rice. Hydrolyzed (**d**) brown rice, (**e**) corn, and (**f**) white rice [67]. (Reprinted with permission from Govindaraju et al., 2020.)

Optical microscopy has been a part of biopolymer research for a long time to analyze samples, due to its simplicity and little-to-no sample preparation. Optical microscopy assists in observing different features such as size, shape, uniformity, void content, failure analysis, and quality control [98,99,100]. An optical microscope can discern the filler dispersion in composites on a larger scale, thus providing a wider perspective of filler distribution globally in the sample than other techniques. It has been successfully used in determining the uniformity of poly(ε-caprolactone)/chitosan blend fibers to be used in tissue engineering [65], wherein the dry fiber diameter as a function of total polymer concentration was analyzed and the average fiber diameter was determined by utilizing the microscopy images with a computer image analyzer [65]. Similarly, optical microscopy has been used to analyze the microstructure and morphology of starch granules assisting in determining the performance of the composites as a function of the size and shape of the granules [66]. Results acquired via an optical microscope with 50× magnification revealed that the granules had a variety of morphology from oval, spherical, polygon, to irregular shapes depending on their botanical origin [66]. Govindaraju et al. made similar observations, where they observed varied morphology of starch granules obtained from different sources (Figure 3); besides evaluating the granule sizes and observing starch degradation after hydrolysis [67], the granules were found to be polyhedral in the case of rice starch, while corn starch showed spherical and polyhedral structures.

The optical microscopy technique has also been able to discern the phase separation between different components and the crystallization behavior of different biopolymers [101,102]. Herein, microscopes equipped with a temperature controller have been used to study the effect of temperature and blend ratio on the phase structure of the ensuing biopolymer blends. Micrographs of the blend systems were obtained in situ to study the crystallization behavior of the blends [101,102]. Optical microscopy is not only limited to solid samples, and has been used to analyze emulsions of biopolymers to determine the size shape and uniformity of droplet size [103,104]; images are obtained by placing a drop of emulsion on the microscopic slide and covering it with a coverslip before observing under the microscope.

When the dimensions of the samples fall into the nanometer scale like in the case of cellulose nanocrystals and cellulose nanofibers, the use of optical microscopy might seem impractical. However, as mentioned earlier there have been massive developments in the field and many variations of optical microscopy are available nowadays; fluorescence microscopy technique has broadened its horizons by extending the diffraction-limited resolution to nano dimensions and achieving super-resolution. The resolution is extended to smaller values by employing far-field imaging methods like confocal, multiphoton, 4Pi microscopy, and structured illumination and spatially patterned excitation to achieve super-resolution in florescence spectroscopy [105,106,107]. To use fluorescence spectroscopy on the samples, they must contain chromophores and there have been different approaches to label either the matrix or the filler with chromophores. An example of such a study is where cellulose nanocrystals of varying charge contents were fluorescently labeled with 5-(4,6-dichlorotriazinyl) aminofluorescein (DTAF) and analyzed [108]; labeled nanocrystals were fluorescently active and were comparable with the unlabeled counterparts in surface chemistry and behavior. The labeled nanocrystals were used as optical markers to determine the dispersion quality of cellulose nanocrystal loaded polyvinyl alcohol composites [108].

Laser scanning confocal microscopy (LCSM) is another variant that presents certain advantages over the conventional wide-field optical microscopy, as it has the ability to minimize or eliminate the background noise from the focal plane and is capable of taking a series of optical sections in case of thick specimens. It uses spatial filtering techniques to remove any out-of-focus light from specimens with a thickness exceeding the immediate focus plane. This technique can provide better images than conventional fluorescence microscopy from the samples prepared for the same, but LCSM cannot provide nanoscale resolution [109,110]. LCSM, in combination with Förster resonance energy transfer (FRET), has been successfully used in the study of DTAF labeled cellulose nanofibrils (CNF) incorporated into coumarin 30 (C30) labeled polyethylene (PE) matrix [111]. FRET can be defined as a phenomenon wherein an energy transfer occurs between a donor chromophore and an acceptor chromophore when certain conditions are met. FRET enables certain nano features, which are not in the resolution limit of the optical microscopy, and the FRET/LCSM combination can provide information at nanoscale while scanning the entire sample at macroscale. This technique has been used to extract information about the interface of the CNF and the PE matrix.

**Figure 4:**
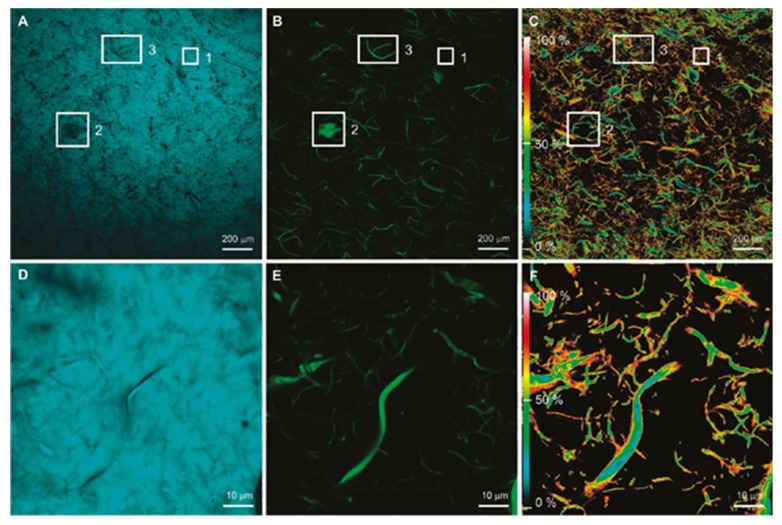
(**A**) Coumarin 30 (C30) fluorescence (donor filter set) at 5× magnification; (**B**) cellulose nanofibril (CNF) fluorescence (acceptor filter set) at 5× magnificationl (**C**) energy transfer efficiency map at 5× magnification; (**D**) C30 fluorescence at 100× magnification; (**E**) CNF fluorescence at 100× magnification; (**F**) energy-transfer-efficiency map at 100× magnification [111]. (Reprinted with permission from Zammarano et al., 2011.)

From Figure 4, one can see images with the fluorescence of C30 [(A), (D)] and CNF [(B), (E)] obtained by donor and acceptor filters while the energy transfer efficiency maps can be seen in Figure 4 [(C), (F)], which were calculated by applying algorithms to the confocal images. The inset images evidently show that FRET does not occur when there is no distribution of CNF in the PE matrix, which can be explained by the inability of the C30 to penetrate the CNF agglomerates. All this information suggests that information in nanoscale related to the interface of the filler and matrix can be obtained by LCSM/FRET.

From all the above-mentioned examples and studies, it is evident that even though the optical microscopy technique is one of the oldest techniques it can still provide a significant amount of information; variants of optical microscopy are competent with the newer techniques and will prove to be useful for a very long time.

### 3.2. Scanning Electron Microscopy

Scanning electron microscopy (SEM) is one of the most versatile, distinguished, and popular techniques in research as well as industrial sectors. It is a class of electron microscopy which uses a high-energy electron beam to scan the samples and provide a high magnification and resolution image. The electron beam from the electron gun interacts with the electrons on the sample and produces certain signals about the surface topography. The SEM images are obtained by analyzing the signals from the secondary and backscattered electrons, which contain information regarding the sample. This technique provides abundant information about the samples being analyzed, including but not limited to surface morphology, crystallinity, elemental composition [112]. In theory, when the electron wavelength and their energy are taken into account, resolutions smaller than the radius of an atom could be obtained by this technique. However, limitations arising due to the lenses and the sample preparations restricts its working resolution to the order of 1–2 nm [112]. The SEM analysis can be carried out under high vacuum or low vacuum and even under wet conditions [113]. Samples for the analysis of SEM are typically frozen under liquid nitrogen and coated to avoid charging and metal shadowing or common deployment of negative staining for contrast enhancement [114].

**Figure 5:**
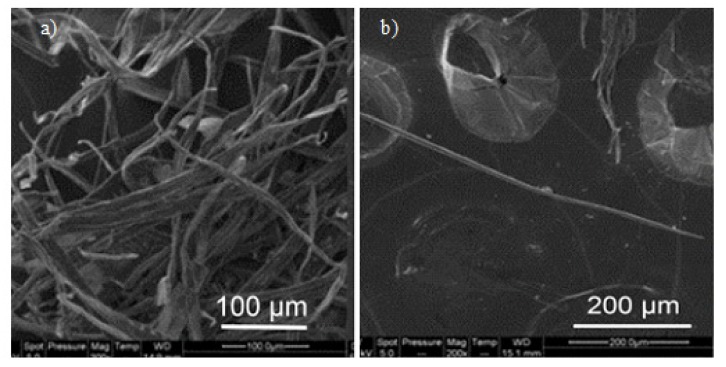
Scanning electron microscopy (SEM) images of different cellulose fibers; (**a**) bundles of unbleached fibers; (**b**) individual bleached fibers [81]. (Reprinted with permission from Nagalakshmaiah et al., 2016.)

SEM has been widely used for decades in the study and analysis of biopolymer systems to gain information pertaining to the structure, morphology, size, shape, surface modifications, wear and tear, etc. SEM studies have played pivotal roles in the analysis of biopolymers [115,116,117], biopolymer nanoparticles [118,119], biopolymer assisted nanoparticles [21,22,23], biopolymer nanocomposites [120,121,122,123,124], and biopolymer fibers [125,126,127,128]. SEM images can be helpful in observing and analyzing different particle structures varying from fibers to microparticles and nanocrystals [129,130]; SEM provides an effective way to analyze these particles and their surfaces, and a medium to measure the particle size and diameter as well [81] (Figure 5).

**Figure 6:**
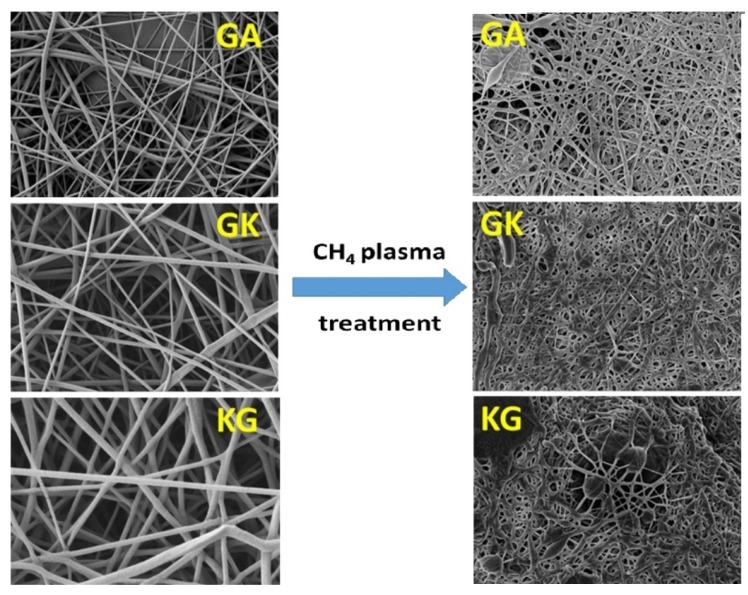
SEM images of polyvinyl alcohol (PVA)/gum Arabic (GA), PVA/gum Karaya (GK), and PVA/Kondagogu gum (KG) before and after plasma treatment [19]. (Reprinted with permission from Padil et al., 2016.)

SEM imaging is widely used to determine the surface topography, homogeneity, and any phase separation between different components in a biopolymer and composite film [116]. These properties have a direct correlation with the mechanical properties of the film, which in turn determines its end-use applications. SEM studies on electrospun nanofibers from natural polymers were helpful in determining the diameter and length of the fibers [19] and could distinguish the change in surface topology arising from the plasma treatment; untreated nanofibers had a smooth surface while after plasma treatment SEM micrographs showed significant surface roughness (Figure 6) [19]. In the case of porous structures like scaffolds or membranes, SEM micrographs assist in the determination of pore size, structure, and density [131,132,133].

**Figure 7:**
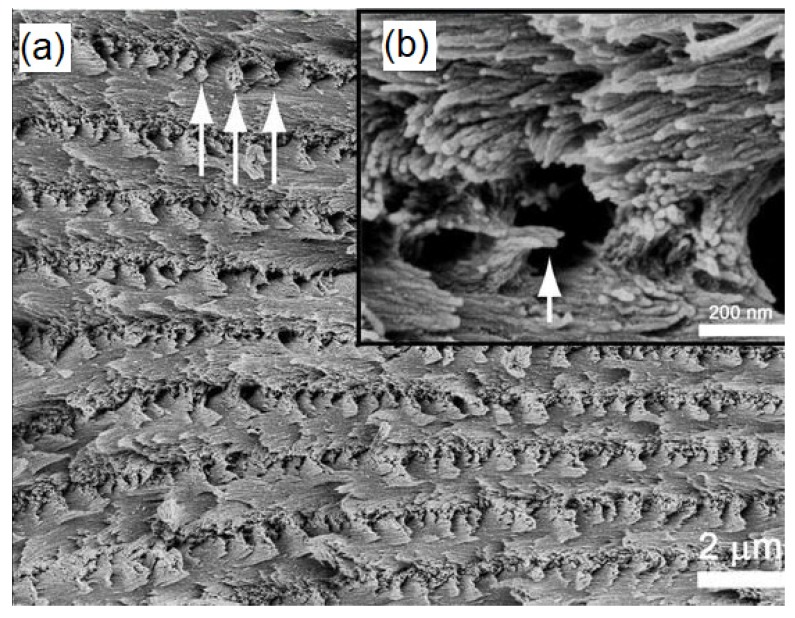
SEM images of the fractured surface across the cellulose nanocrystal (CNC) film (**a**) High magnification image of the layered structure of the film, (**b**) Cross-sectional image showing (inset Figure b) the helicoidal arrangement of nanocrystals. [70]. (Reprinted with permission from Majoinen et al., 2012.)

In nanocomposite characterization, SEM is primarily used to determine the dispersion and distribution of the fillers in the polymer matrix. In addition, SEM images also show the presence of any agglomeration of the fillers or additives within the polymer matrix [134,135]. Intercalation and exfoliation of layered nanoparticles, such as montmorillonite within the soy protein isolate nanocomposites, have been predicted by analyzing the SEM images [136,137]. In addition to the surface morphology, SEM has been successful in observing the alignment of the crystals in cellulose nanocrystal (CNC) films [70]; high magnification SEM image of a fractured cross-section of the CNC films revealed that the direction of the chiral nematic axis changes with the location in the film. Figure 7a shows a high magnification image of the layered structure of the film while the fan-like structures seen in Figure 7b correspond to the cross-section of a left-handed helicoidal arrangement of the nanocrystals, wherein the helicoid axis is from top to bottom of the image. The results obtained from the SEM images can help in determining the failure mechanisms of the chiral nematic model [70].

Field emission scanning electron microscopy (FESEM) is a variant of conventional SEM, which provides higher resolution images and a greater energy range. One of the main differences between SEM and FESEM is that the latter uses a field emission gun as an electron generation system. This provides highly focused electron beams, which improves spatial resolution and enables the analysis of samples at low potentials; FESEM has already been used efficiently instead of SEM to obtain high-resolution images of biopolymer systems [18,138,139,140,141]. An environmental scanning electron microscope (ESEM) is another variant of SEM, which unlike conventional SEM does not require any special sample preparation like coating, and can examine the specimen at various temperature regimes. ESEM can operate in gaseous atmospheres like in air, nitrogen, argon, oxygen, and even water vapor, thus making it possible to carry out ‘wet imaging’ of samples. ESEM has the ability to analyze dynamic phenomena such as crystallization, wetting, swelling, drying, melting, freezing, as well as material deformation [71,142,143,144,145]. Rizzieri et al. studied the strain deformation and failure behavior of biopolymer gel mixture through in situ ESEM [71]. From the images acquired by ESEM, they carried out the dynamic observations of the microstructure response to mechanical deformations in biopolymer gel mixture systems that were kept hydrated throughout the whole experiment.

**Figure 8:**
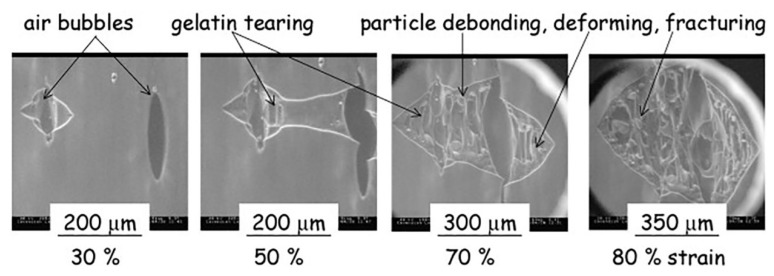
Environmental scanning electron microscope (ESEM) micrographs following the continuous crack propagation on the surface gelatin/maltodextrin sample [71]. (Reprinted with permission from Rizzieri et al., 2003.)

In this study, phase-separated gelatin and maltodextrin composite gels comprising of spherical maltodextrin phases inside a continuous gelatin matrix were subjected to increasing applied strain. ESEM micrographs were taken at a frame refresh rate of 0.5 fps and the applied strain rate was in the order of 10 µm s^−1^. Figure 8 shows that a crack was initiated from an existing defect on the surface, which acts as a stress concentration point. This crack propagates revealing the underlying maltodextrin particles and the fibrillation of gelatin matrix around the particles until the complete failure of the sample is seen from the micrographs.

**Figure 9:**
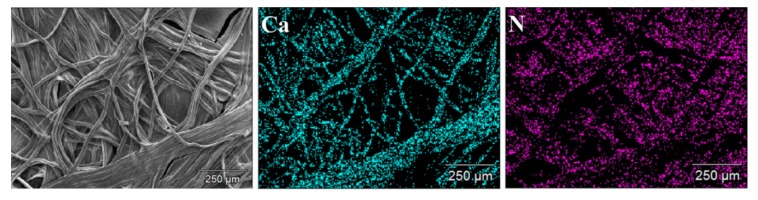
Energy-dispersive X-ray (EDX) mapping images of carboxymethyl chitosan and calcium alginate composite dressings [152]. (Reprinted with permission from Gao et al., 2019.)

Energy-dispersive X-ray spectroscopy (EDX) is an additional accessory included in SEM which functions as an analytical technique to determine the elemental composition or chemical characterization of a sample [146]. EDX works on the basis that every element shows a unique set of peaks on its X-ray spectrum corresponding to its unique atomic structure. A high-energy incident beam excites an electron in an inner shell, and it will be ejected from the shell creating a hole in its place. Another electron from a higher energy outer shell will fill this hole and the energy difference between the shells will be released in the form of X-rays which will be characteristic of the atomic structure of the emitting element [146]. EDX studies on biopolymer systems help in determining the elemental composition, impurities, chemical modifications, and functionalization of the samples [147,148,149,150]. EDX analysis can be very helpful in analyzing biopolymers with chemical modification as this enables researchers to determine the presence of heteroatoms (chlorine or sulphur) used in the modification [151]. EDX assists by providing the elemental composition of the analyzed material and EDX mapping can provide elemental mapping in the analyzed sample. In addition to providing the elemental composition and content, it helps in the analysis of dispersion and distribution of compounds within the sample. For example, EDX mapping can be helpful in determining the distribution of carboxymethyl chitosan and calcium alginate in a composite by analyzing the N element content of chitosan and Ca element of calcium alginate (Figure 9) [152].

### 3.3. Transmission Electron Microscopy

A transmission electron microscope (TEM) is one of the most powerful microscopes available today which is used as an analytical tool to analyze and visualize the samples in the realm of a nanoscale [153,154]. TEM and light microscopes both operate on the same basic principles; however, the major difference between them is that TEM uses electrons instead of light. Since the wavelengths of electrons are much smaller when compared to that of light, images obtained from TEM have much higher optical resolution than those of a light microscope. This enables TEM to reveal the tiniest details of the sample, sometimes as small as individual atoms. The name ‘transmission’ meaning, ‘to pass through’ is essentially how the TEM operates, by passing a beam of high-energy electrons through very thin samples [155]. The electron beam may not pass through some sections of the sample and can be deflected by some sections. The electrons transmitted through the samples are collected from below through a camera or onto a phosphorescent screen to obtain the images. The dark part of the image represents the areas in the sample through which very few or no electrons are transmitted, the brighter part represents the areas through which more electrons are transmitted, and a range of grey color patterns are obtained depending on the way the electrons interact with the sample [153,154]. The most tedious part of TEM analysis is sample preparation, which requires a lot of time as the samples should be very thin in order to transmit sufficient electrons through with minimum energy loss [155]. TEM can be used to obtain information related to particle size, shape, distribution, crystallinity, crystallite size structure, and orientation [156,157,158].

**Figure 10:**
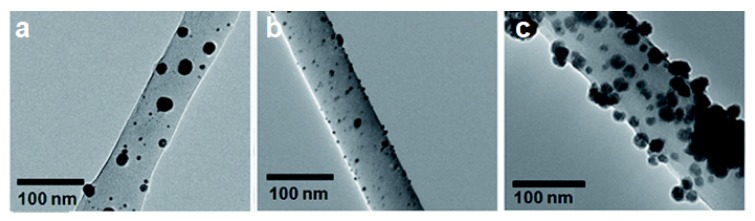
Transmission electron microscope (TEM) image of (**a**) Ag, (**b**) Au, and (**c**) Ni nanoparticles on cellulose nanofiber surface [74]. (Reprinted with permission from Gopiraman et al., 2018.)

TEM is a well-established analytical technique in many fields including but not limited to nanotechnology, material science, medical research, biomedical, biological, and semiconductor research. TEM has found extensive use in biopolymer research over the past decades and has helped reveal information at the atomic scale. TEMs have been known to provide information on the topological, morphological, compositional, and crystalline information of biopolymer systems. In biopolymer nanocomposites, TEM is highly recommended when there is a need for in-depth study to understand the quality of the internal structure, spatial distribution of different phases, and defect structure of nanocomposites. It has been used in studying the dispersion of nanoparticles within the matrix. As can be seen from Figure 10, the TEM images clearly show the nanoparticle dispersion on cellulose nanofiber. Further magnification allowed for the confirmation of the metal nanoparticle attachment onto fiber surface and assisted in calculating the average particle size [74].

**Figure 11:**
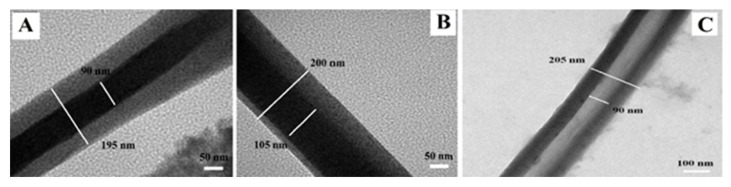
TEM images of PEO-chitosan biocomponent electrospun fibers showing (**A**) concentric core-shell structure, (**B**) eccentric core-shell structure, and (**C**) hollow chitosan shell structure [75]. (Reprinted with permission from Pakravan et al., 2012.)

TEM has been an invaluable asset in characterizing core-shell and hollow nanoparticles and nanofibers by observing the contrast created by the electron beam diffraction, which represents the distinctive phases present in the analyzed sample. TEM images of core-shell structured polyethylene oxide (PEO)-chitosan electrospun fibers depicted in Figure 11 reveal two distinct phases [75], where the dark region represents the core while the bright region represents the shell structure. Further, TEM assists in the identification of different concentric and eccentric structures as well as in the measurement of diameters of the core and the shell components of the fiber. Additionally, the fibers once washed with water to remove the PEO core and the TEM analysis of the ensuing hollow structure can confirm the complete removal of the PEO core while the chitosan shell maintains its physical structure [75]. High-resolution transmission electron microscopy (HRTEM) is a variant of TEM with resolutions below 0.5 Å [159] which facilitates the imaging of specimens at an atomic scale and enables the analysis of the atomic structure of the samples [159]. HRTEM has been an invaluable asset in the analysis of biopolymer-assisted formation of nanoparticles including the study of their crystal planes [160,161,162], the crystal structure of cellulose [163], nanocomposites [164,165], and even in the molecular orientations of biopolymers [166].

Conventional TEM utilizes electron beams in high vacuum conditions, but certain materials are not compatible with these high vacuum conditions. In such cases, a specialized variant of TEM called cryo-TEM is employed for the analysis of the samples [167,168] where samples are often frozen by rapidly plunging into a liquid ethane bath for preservation purposes. The solvent around the specimen will be frozen upon dipping in the cold medium thus ensuring the cryogenic preservation of the specimen. Water or salt solutions are commonly used as solvents to ensure the stability of the samples. However, care must be taken to expedite the process of freezing the sample very quickly, as this will prevent the formation of cubic ice, which interferes by absorbing the electron beam and obscuring the sample. There are many advantages associated with cryo-TEM including the analysis of the sample in vitreous ice, which actually preserves the structural information largely and reflects the state of the sample prior to freezing. In addition, since the sample is not exposed to any adhering surface, the shape observed is the true shape of the sample, which is not affected by any kind of attachments that might result in flattening. Cryo-TEM is also useful to analyze nanoparticle suspensions, which prevents any changes to the sample induced by drying or staining such as agglomeration, degradation, deformation. Several researchers have utilized cryo-TEM to study cellulose nanocrystals in its native state, as they tend to agglomerate upon drying [169,170]. Scanning transmission electron microscopy (STEM) is a technique which combines the operational modes of both SEM and TEM [171] and operates by focusing a convergent electron beam onto a small area of the sample. To obtain an image, the electron probe raster scans thereafter propagate through the sample [172,173]. STEM imaging could be done by using secondary or backscattered electrons, however, superior spatial resolution and better signal levels are obtained by recording the transmitted electrons [172,173]. Several detectors are employed in the STEM and each of them provides a distinct complementary view of the sample. Electrons transmitted through the sample in a path close to the optical axis are collected by a bright field detector so the holes appear bright, whereas an annular dark field or a high-angle annular dark field detector (HAADF) collect the scattered electrons excluding the transmitted beam so the holes appear dark [172,173]. This ensures the recording of maximum possible details about the sample from each scan. STEM has been used in the analysis of biopolymers and biopolymer nanocomposites. The bright field and dark field imaging of the STEM has assisted in the determination of particle size, shape, filler dispersion and agglomeration, and biopolymer coatings as well [174,175,176,177,178,179,180,181,182,183]. The HAADF-STEM images obtained for metal oxide nanoparticles stabilized in carboxymethyl-cellulose (CMC)/cetyltrimethyl-ammonium-bromide (CTAB) templates have assisted authors to distinguish between the two phases [179]. The metal oxide nanoparticles, seen as brighter zones, are found to be embedded in the darker zones of CMC/CTAB templates. Furthermore, the bright field and HAADF images have assisted in determining experimental parameters controlling the size and morphology of the nanostructures [179].

**Figure 12:**
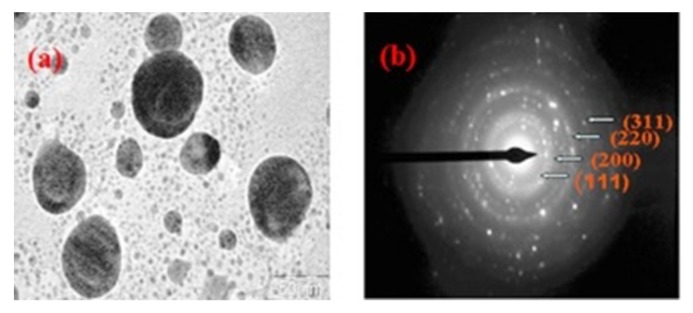
TEM micrograph of (**a**) guar gum capped silver nanoparticles; (**b**) selected area electron diffraction (SAED) pattern [192]. (Reprinted with permission from Vanamudan et al., 2016.)

Selected area electron diffraction (SAED) is a crystallographic analysis technique often accompanied by TEM. The majority of the TEM equipment, having a parallel electron beam source, are capable of carrying out the SAED analysis, which provides diffraction patterns as a result of the electron beam scattering by the sample lattice. The electrons elastically scattered by the lattice obey Bragg’s law and hence it is possible to index the diffraction spots in the pattern and in turn identify the phases and analyze the sample structure. SAED aperture has the capability to select and analyze 0.5–1 µm length of the sample; therefore, it is possible to selectively analyze the sample. This is helpful while analyzing polycrystalline samples because when more than one crystal contributes to the diffraction pattern it can be difficult to analyze the diffractogram. One can obtain information regarding the structure of the sample including the crystalline symmetry, crystal defects, unit cell parameter, and texture of the sample. SAED has been used in the analysis of biopolymers to obtain information regarding their crystalline structure [184,185,186,187]. However, SAED is proven to be a more prominent technique in the analysis of the biopolymer-assisted synthesis of metallic nanoparticles [22,23,188,189,190,191]; SAED analysis of biopolymer gum guar capped silver nanoparticles revealed information about the crystalline structure of the nanoparticles. The diffraction pattern (Figure 12) consisted of concentric rings with bright dots, which suggests that the synthesized particles are highly crystalline in nature; the rings were further assigned to (111), (200), (220), and (311) planes of the face-centered cubic structure of the particles [192].

### 3.4. Scanning Probe Microscopy

Scanning probe microscopy (SPM) encompasses a family of several unique techniques, which are capable of imaging not only the nanoscale structures and surfaces but atoms as well [193]. The main distinguishing feature that sets apart the scanning probe microscopy from other techniques is that it uses no lenses but a probe to interact with the sample [193]. The probe moves over the sample surface and a computer gathers and analyses this data to create an image of the surface. The probe, which is as sharp as an atom, is usually mounted at the end of a cantilever and is moved over the surface precisely. During the scanning process, different forces, including electrostatic forces, magnetic forces, mechanical contact, Van der Waals interactions, chemical bonding, and capillary forces, can deflect the cantilever tip and the SPMs are capable of measuring these deflections. A laser, which is focused onto the cantilever, is reflected off its top onto a detector and measures the vertical deflection caused by any of the above-mentioned forces [194,195]. Multifarious interactions are studied and analyzed depending on the probe sensors being used and the interactions being measured between the probe and the sample surface determines the variant of SPM being used [194,195]. For example, atomic force microscopy (AFM) measures electrostatic forces, magnetic force microscopy (MFM) measures magnetic forces, chemical force microscopy (CFM) measures chemical interactions, while scanning tunneling microscopy (STM) measures electric current flowing between the probe and sample surface [196]. A wide variety of techniques fall under the family of SPMs, herein, the major techniques used in biopolymer field are discussed.

(a) Atomic Force Microscopy

Atomic force microscopy is a distinguished analytical tool with a capacity to determine surface structures with high spatial resolution [197,198]. An AFM operates on the basic principles of SPMs wherein it analyzes the sample surface by means of a very sharp tip, which is often a 3–6-micron long pyramid with a diameter of less than 100 Å. The tip is present at the free end of a 100–200 µm long cantilever, which undergoes deflection/bending due to the forces between the probe and the sample [199,200,201]. AFMs are capable of measuring these lateral or vertical deflections by means of an optical lever that reflects a laser beam off the cantilever [197]. A position-sensitive photodetector detects these deflections and allows a computer to generate an image of the surface topology of the sample being analyzed. Over the years, like any other technique, many variations have been developed for AFM to suit modern needs and they are applicable to all the samples, however, not all variants yield the desired quality results. Proper use of these variants enables one to study and analyze the samples at fundamental, even at the atomic level. The three most popular modes employed in AFM are contact mode, non-contact mode, and tapping mode.

In contact mode, as the name suggests, the tip is in constant contact with the sample. Contact mode further has two subclasses, namely constant height mode and constant force mode. In constant height mode, the height of the scanner is fixed during the scanning process and the spatial variations of the cantilever tip deflection are used to generate the topographic image. This technique is used when measuring samples with atomically flat surfaces to obtain atomic-scale images and is capable of quick scans [202]. While in the constant force mode, the cantilever deflection is kept constant and hence the force on the sample is constant. A feedback circuit is used to move the sample or the tip up and down to keep the deflection constant, and measuring the z-movement provides the changes in heights in the sample. As the tip will be in constant contact with the sample, there is a chance of destruction of samples due to frictional forces [203]. In the non-contact mode, the cantilever tip will not be in contact with the sample surface but will be hovering above it at a very close distance of 5–10 nm. In this mode, the cantilevers used will have high spring constants and have the advantage of having negligible frictional forces. This is very helpful in analyzing the soft samples without altering their surface characteristics [204]. A major drawback of this mode is that when compared to the contact mode it has very low lateral and z-resolution, which is circumvented by employing the tapping mode or intermittent contact mode, wherein, instead of hovering, the cantilever tip vibrates and scans above the surface and momentarily makes contact with the sample surface. The amplitude of vibrations decreases, and a phase shift occurs because of the interactions of the cantilever tip with the surface of the sample. A feedback circuit can be used to move the sample or the tip in the z-direction and to keep the amplitude or the phase shift constant. This mode requires stiffer cantilevers with very small damping factors. Due to its high-resolution imaging and close to non-destructive nature, as well as its applicability in liquid and air conditions, amplitude modulation mode is the most popular mode of AFM [205].

**Figure 13:**
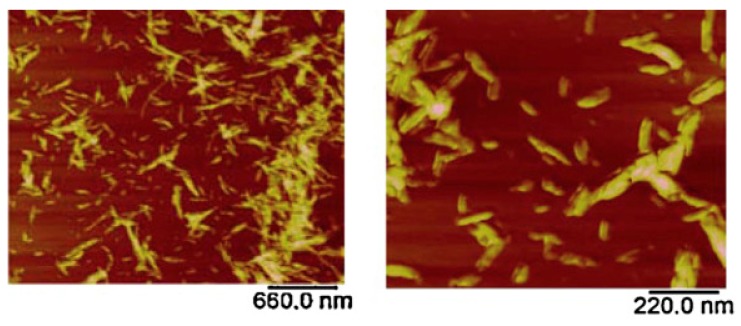
Atomic force microscopy (AFM) images of cellulose nanocrystals at **a**) low and **b**) high magnification. The length, width, and aspect ratio were 90–180 nm, 4–6 nm, and 26, respectively [81]. (Reprinted with permission from Nagalakshmaiah et al., 2016.)

AFM has been deployed in the field of biopolymers for quite some time where it has been used in the analysis of complex structures formed by the association of biopolymers. AFM has been a powerful technique in the analysis and understanding of molecular interactions, nature, and strength of surface forces in the biopolymer systems. Previously, when AFM was first introduced in the biopolymer field, the imaging was carried out in air by depositing biopolymers on mica substrates. This technique works well for stiffer samples [206,207]; however, for soft samples, the results obtained by this technique were unreliable due to the interference of residual water present on the tip and the sample surface [208]. This residual water resulted in adhesive forces causing damage or displacement of molecules and was overcome by employing contact mode imaging in liquids [209], tapping mode [210], and non-contact imaging [211] modes. AFM has been used in the analysis of single molecule [76], conformations [78], local structure and elasticity of gels [212], gelation mechanisms [213], and nanoparticle topography [79,80]. Quantitative information including the contour length and the molecular heights has been measured accurately using high-resolution AFM images. Studies on the single chitosan strands deposited on mica surface revealed the contour lengths of chitosan strands around 94–178 nm with an average molecular height of 0.45 ± 0.04 nm [214]. Similar studies have been conducted to determine the size of a single cellulose nanocrystal; AFM has provided an efficient way to evaluate the length, width, and aspect ratios of these individual crystals (Figure 13) [81].

The difference in the molecular structure of the biopolymers has also been determined by the AFM technique; the molecular structure of xanthan biopolymer produced by several different strains of Xanthomonas campestris was studied by AFM [77].

**Figure 14:**
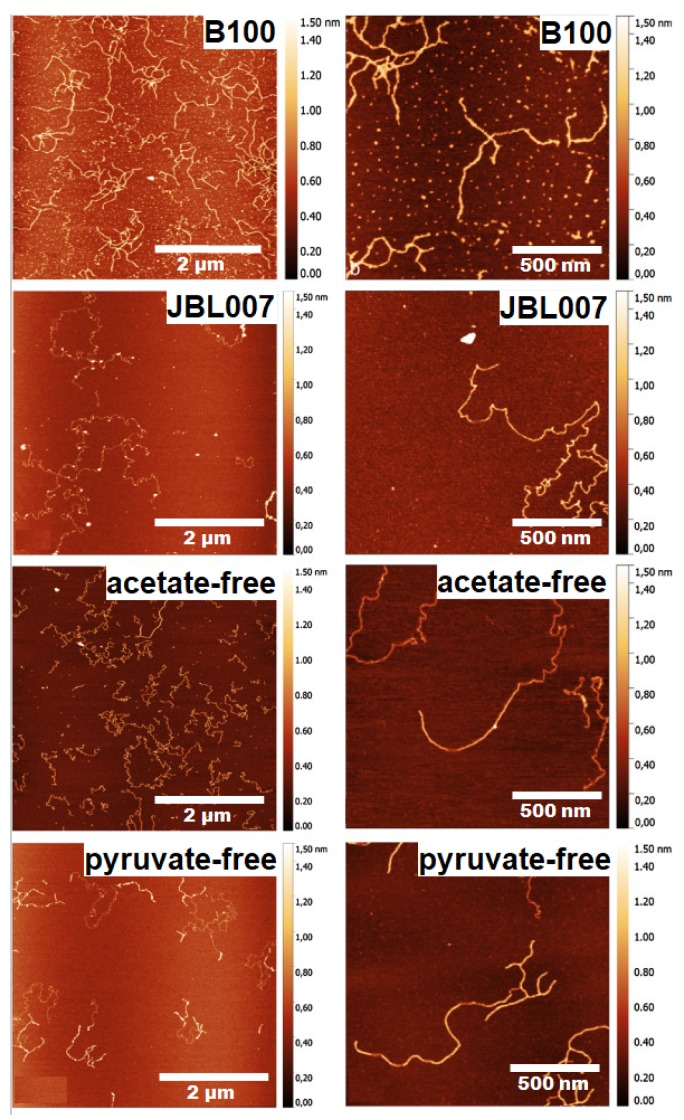
AFM topographical images of xanthan from different sources. Images left provide the overview while right show the detailed structure of the samples [77] (Reprinted with permission from Teckentrup et al., 2017.)

The AFM images depicted in Figure 14 revealed different structural features for different strains. Xanthan from the wild-type strain Xanthomonas campestris B100 showed branching and overlapping sections while the one from the strain JBL007 showed no branching. The acetate-free xanthan shows no branching with single and double-stranded areas and the pyruvate-free xanthan show a homogenous and branched structure with single and double strands (Figure 14).

(b) Chemical Force Microscopy

**Figure 15:**
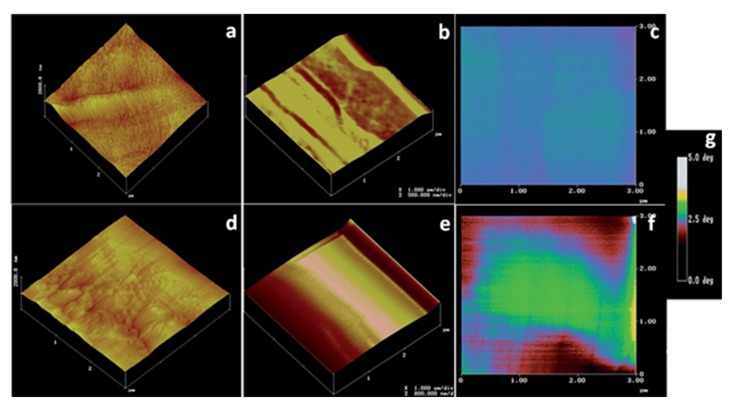
(**a**) Tapping mode image of cellulose isolated from switch-grass; (**b**) topographic image of switch-grass cellulose obtained in force-volume mode; (**c**) adhesion force image of switch-grass cellulose obtained in force-volume mode; (**d**) tapping mode image of extractives-free switch-grass; (**e**) topographic image of extractives-free switch-grass obtained in force-volume mode; (**f**) adhesion force image of extractives-free switch-grass obtained in force-volume mode; (**g**) a false color scheme based on the calibrations used for the depiction of the adhesion forces [222]. (Reprinted with permission from Lee et al., 2015.)

Chemical force microscopy (CFM) is a form of AFM, which relies on the modification of probe tips by functionalization to introduce chemical specificity into the measurements [215]. It is a powerful technique for the quantitative analysis of intermolecular interactions between distinct chemical functionalities [215,216]. CFM detects chemical bonding forces between functional groups on the surface of a specimen by attaching ligands on to the probe tip [215,216]. The principle involves bringing a chemically modified tip in contact with the sample surface and measuring the resulting attractive or repulsive forces as the tip is approached or withdrawn from the sample [217,218]. The measured forces are then mapped and compared to the surface structures detected by the topological imaging for further analysis. These measurements are proven to be helpful in identification purposes, to determine compatibility between two materials, and to predict interactions between materials [219]. CFM has proven its use in the analysis of biomaterial and biopolymer fields, and has been used in the characterization of cellulose [220], chitosan [82], and DNA [221]. Lee et al. analyzed the cellulose in biomass samples through CFM by functionalizing the silicon nitride cantilever probes with triethoxysilyl *N*-propyl gluconamide [222]. AFM images obtained by tapping mode, topographic force-volume mode and their corresponding adhesion force measurements, along with the false color scheme for measuring adhesion force strengths are depicted in Figure 15. AFM images of switch-grass cellulose obtained by tapping mode showed micro-fibrils over the entire sample and the images obtained by topographic force-volume mode with lower spatial resolution showed parallel microfibril bundles. The corresponding adhesion force-mapping image revealed a uniform adhesion force throughout the sample. In the case of extractives-free switch-grass cellulose, the tapping mode images revealed a heterogeneous structure with aligned fibrillary structure along with less defined globular masses embedded in the hemicellulose and lignin matrix. The topographic force-volume mode images showed only general contours; however, the adhesion force image shows areas with adhesion force similar to that observed for cellulose microfibrils bound by regions of low adhesion probably the lignin and hemicellulose as observed in tapping mode [222]. All this information obtained from CFM suggests that it is an invaluable asset in the microscopical analysis of biopolymers.

(c) Magnetic Force Microscopy

Magnetic force microscopy (MFM) is another variant of AFM, which is capable of analyzing surfaces with magnetic properties at the nanoscale [223,224]. In MFM, the surface of the sample is scanned by means of a standard tip coated with a ferromagnetic film of a few nanometers thickness [223,224]. The operating mechanism of MFM is very similar to that of AFM with both static and dynamic modes of analysis available, but the dynamic mode is more popular as it offers higher sensitivity. While scanning the specimen with the tip of the cantilever, the magnetic forces between the sample and the tip cause the cantilever to bend and the oscillations are recorded. However, while scanning the specimen, along with the magnetic forces the Van der Waals forces will also be present. This can be controlled by regulating the distance of the tip from the sample as the effect of Van der Waals forces wear off at longer tip-to-sample distances while the magnetic forces persist [225]. Van der Waals forces are used to obtain the topographical image of the surface as the forces vary with the tip-to-sample distance. In general, for successful MFM imaging, the sample is first scanned in the close range where the Van der Waals forces are dominant to acquire a topographical image and then the tip is lifted to a region where the magnetic forces are dominant and scanned for MFM image. This technique is advantageous as it minimizes the effects caused by non-magnetic forces and ensures only the record of magnetic forces [226,227].

**Figure 16:**
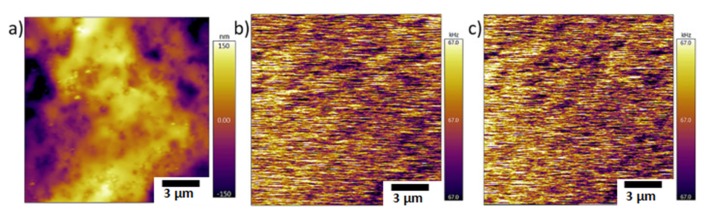
Magnetic force microscopy (MFM) images of pure gelatin films (**a**) topographical image; (**b**) frequency image at 0 Tl (**c**) frequency image at 0.47 T; both frequency images are identical indicating no response to external magnetic field [228]. (Reprinted with permission from Marín et al., 2018.)

The use of MFM in biopolymer systems is marginally explored and is limited to the analysis of magnetic biocomposites [83,228,229,230,231]. The magnetic properties of the magnetic nanoparticles loaded biopolymeric systems can be evaluated by means of MFM. Since the biopolymers have no response to the external magnetic field (Figure 16) [228], MFM provides information regarding the dispersion and encapsulation of magnetic nanoparticles inside the biopolymer matrix [83]. The aggregations of magnetic nanoparticles can also be determined by measuring the variation in frequency shifts vs. the position of the tip [228].

### 3.5. Scanning Tunneling Microscopy

A scanning tunneling microscope (STM) is a non-optical microscope, which consists of an electric probe tip that is used to scan over a sample surface at a constant spacing. STM operates on the principle of quantum mechanical phenomenon called tunneling, which occurs when the wave-like properties of electrons allow them to pass through a barrier that, in general, they should not be able to pass through. The effect of tunneling reduces as the gap between the two surfaces increases. To analyze a sample by STM, it must be capable of conducting electricity. The extreme end of the tip is very sharp, down to a single atom, and a voltage is applied between the tip and the sample surface, resulting in the tunneling of electrons. As the electrons begin tunneling, a current starts flowing and this current can be measured. When the probe is moved over the surface of the sample, variations in the tunneling current corresponding to the surface structural changes are observed. A feedback circuit is employed to monitor and make necessary changes to maintain a constant tunneling current, which is recorded and processed by a computer to provide a topological image of the sample surface. There are two modes in STM—constant current mode and constant height mode. In the constant current mode, a feedback loop adjusts the height to keep the current constant and the image of the sample surface is generated by recording the vertical position of the tip. In the constant height mode, the vertical position of the tip remains unchanged and the change in current as a function of the position is recorded to obtain the topographical image.

**Figure 17:**
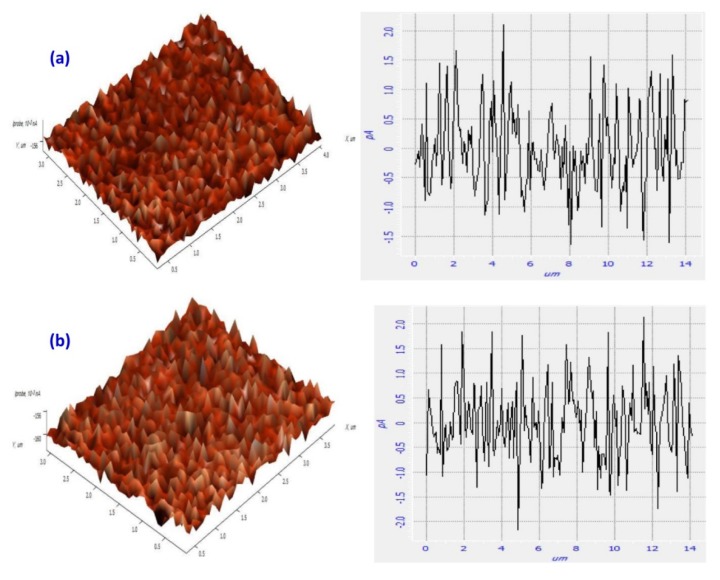
Scanning tunneling microscopy (STM) images and height profiles of methylcellulose-based polymer electrolyte (**a**) before H_2_S treatment, (**b**) after H_2_S treatment [87]. (Reprinted with permission from Abdullah et al., 2018.)

STM has a high resolution of 1 Å in the sample plane and as high as 0.1 Å in the vertical plane, and is capable of imaging at ambient pressures and in liquids with minimum specimen damage. Despite these advantages, the use of STM in the analysis of the biopolymer field is limited by poor conductivity, instability, flexible elasticity of biopolymers. However, these drawbacks have been overcome by imaging carbon cast replicas [232], STM related AFM [233], and by conductive coating [234,235]. The use of STM in the biopolymeric field is very limited and has been used in the analysis of the molecular structure of bacterial polysaccharides like gellan gum and xanthan gum, deposited on highly oriented pyrolytic graphite (HOPG). The STM images of xanthan gum deposited from a high concentration aqueous solution onto HOPG revealed stiff aligned rod-like molecules [84]. In the case of gellan gum, the double-helical structure forming into cation-mediated aggregates was observed in the STM images [85], suggesting that STM has the ability to analyze intricate details related to the molecular structure of biopolymers. STM has been further used in the studies of cellulose crystalline fibrils [86], surface modification of methylcellulose [87], biocomposites [236], collagen [237], and other polysaccharides [238]. Abdullah et al. studied the surface modification of methylcellulose/cobalt nitrate polymer electrolytes by H_2_S, and the STM analysis of samples revealed an increase in surface roughness of the samples post H_2_S treatment (Figure 17) [87]; maximum height and root mean square deviation of the roughness profile were increased to 4.296 and 0.752 pA from 3.734 and 0.705 pA, respectively. This increase in surface roughness was attributed to the formation of CoS nanoparticles, which were dispersed homogenously throughout the film as indicated by the relatively low value of root mean square of the gas treated samples.

## 4. Conclusions

The emergence of new biopolymer-derived materials creates a need to gain insights into their complex structure and morphology. The influence of the micro and nanostructures of biopolymers on the macroscopic scale dictates the need for their study as these structural and morphological variations influence the physical and mechanical properties of the final material. In order to understand new material, it is crucial to correlate the structural observations with the intrinsic material properties as microscopic analysis can obtain images revealing the intricate details of the microstructures present within the samples. These microscopic techniques are of paramount importance in the analysis of fundamental structures, morphology, surface properties, molecular structure, microstructure, chemical composition, topography, and interfaces. They also provide information related to the dispersion, distribution, intercalation, exfoliation, and aggregation of nanoparticles in the composites. However, care should be taken while interpreting these images. Materials’ intrinsic nature, analysis technique, sample preparation methods can have a significant influence on the final structures revealed in the images. Biopolymers are often sensitive to the electron beams employed in the electron microscopes and can undergo changes or impairments. If these changes are not considered while interpreting the images obtained, the results may not be reliable. Despite providing a plethora of information, sometimes the microscopic techniques can yield ambiguous results. Hence, more often different microscopic techniques are used in conjunction with each other and with other characterization techniques to obtain ‘in-depth’ knowledge and understanding of the material. The advent and development of microscopic techniques such as aberration-corrected electron microscopy and 3D electron microscopy have opened new horizons in the material understanding. These microscopic techniques have served as an indispensable tool in the development and commercialization of biopolymers.

## Figures and Tables

**Table 1:** Summary of various microscopic techniques, their applications and relevant biopolymers illustrated in the current review.

Technique	Application	Biopolymer	References
Optical Microscopy	Fiber diameter	Poly(ε-caprolactone)/chitosan blend	[65]
	Size and ahape	Starch granules	[66,67]
	Filler dispersion	starch/ Gum Arabic/nanocellulose	[68]
Scanning electron microscopy (SEM)	Particle size	Chitosan	[69]
	Particle shape	Starch granules	[66]
	Fiber diameter and surface modification	Gum Arabic, Gum Karaya, Kondagogu gum	[19]
	Crystal alignment	Cellulose nanocrystals	[70]
	Failure behavior	Gelatin/maltodextrin	[71]
SEM + energy-dispersive X-ray spectroscopy	Elemental composition	Cellulose	[72,73]
Transmission electron microscopy (TEM)	Particle dispersion	Cellulose nanofiber	[74]
	Particle Size	Kondagogu gum biopolymer assisted Pt nanoparticles	[24]
	Core shell structure	Chitosan/PEO	[75]
TEM + selected area electron diffraction	Crystallographic analysis	Biopolymer assisted nanoparticles	[21,24]
Atomic force microscopy	Molecular structure and conformation	Xanthan gum	[76,77,78]
	Nanomaterial topography	Nanocellulose	[79,80]
	Particle size and shape	Nanocellulose	[81]
Chemical force microscopy	Chemical interactions	Chitosan	[82]
Magnetic force microscopy	Magnetic properties	Chitosan based magnetic nanohydrogels	[83]
Scanning tunneling microscopy	Molecular structure Particle Size Surface modification	Bacterial polysaccharides Cellulose Cellulose	[84,85] [86] [87]
